# Natural Compounds Such as *Hericium erinaceus* and *Coriolus versicolor* Modulate Neuroinflammation, Oxidative Stress and Lipoxin A4 Expression in Rotenone-Induced Parkinson’s Disease in Mice

**DOI:** 10.3390/biomedicines10102505

**Published:** 2022-10-07

**Authors:** Marika Cordaro, Sergio Modafferi, Ramona D’Amico, Roberta Fusco, Tiziana Genovese, Alessio Filippo Peritore, Enrico Gugliandolo, Rosalia Crupi, Livia Interdonato, Davide Di Paola, Daniela Impellizzeri, Salvatore Cuzzocrea, Vittorio Calabrese, Rosanna Di Paola, Rosalba Siracusa

**Affiliations:** 1 Department of Biomedical, Dental and Morphological and Functional Imaging, University of Messina, 98125 Messina, Italy; 2 Department of Biomedical and Biotechnological Sciences, University of Catania, 95125 Catania, Italy; 3 Department of Chemical, Biological, Pharmaceutical and Environmental Sciences, University of Messina, 98166 Messina, Italy; 4 Department of Veterinary Science, University of Messina, 98168 Messina, Italy; 5 Department of Pharmacological and Physiological Science, Saint Louis University School of Medicine, Saint Louis, MO 63104, USA

**Keywords:** mushroom, neurodegeneration, lipoxin A4, neuroinflammation, oxidative stress

## Abstract

Background: A growing body of research suggests that oxidative stress and neuroinflammation are early pathogenic features of neurodegenerative disorders. In recent years, the vitagene system has emerged as a potential target, as it has been shown to have a high neuroprotective power. Therefore, the discovery of molecules capable of activating this system may represent a new therapeutic target to limit the deleterious consequences induced by oxidative stress and neuroinflammation, such as neurodegeneration. Lipoxins are derived from arachidonic acid, and their role in the resolution of systemic inflammation is well established; however, they have become increasingly involved in the regulation of neuroinflammatory and neurodegenerative processes. Our study aimed at activating the NF-E2-related factor 2 (Nrf2)/heme oxygenase 1 (HO-1) redox system and increasing lipoxin A4 for the modulation of antioxidant stress and neuroinflammation through the action of two fungi in a rotenone-induced Parkinson’s model. Methods: During the experiment, mice received *Hericium erinaceus*, *Coriolus versicolor* or a combination of the two (200 mg/kg, orally) concomitantly with rotenone (5 mg/kg, orally) for 28 days. Results: The results obtained highlighted the ability of these two fungi and, in particular, their ability through their association to act on neuroinflammation through the nuclear factor-kB pathway and on oxidative stress through the Nrf2 pathway. This prevented dopaminergic neurons from undergoing apoptosis and prevented the alteration of typical Parkinson’s disease (PD) markers and α-synuclein accumulation. The action of *Hericium erinaceus* and *Coriolus versicolor* was also able to limit the motor and non-motor alterations characteristic of PD. Conclusions: Since these two mushrooms are subject to fewer regulations than traditional drugs, they could represent a promising nutraceutical choice for preventing PD.

## 1. Introduction

Diseases that impact the central and peripheral nervous systems are referred to as neurological disorders. Such disorders can be caused by nervous system lesions, oxidative stress, inflammation, aberrant protein deposition in neural tissue, autoimmune-mediated neuronal cell death and viral contaminations [1,2]. 

The latest statistics reported in the literature show that following the increase in the life span of the world population, about 80 million people will suffer from dementia by 2040 [3]. Therefore, aging, immune decline and the onset of chronic inflammatory processes are all potential risk factors associated with neurodegenerative diseases including dementia, Alzheimer’s disease (AD) and Parkinson’s disease (PD) [4,5,6,7]. 

Currently, available treatments for neurological disorders focus essentially on symptomatic relief. Therefore, there is a strong need to discover new therapies and neuroprotective agents that can prevent and delay the progression of neurodegenerative diseases [8,9,10,11]. Furthermore, given the undesirable side effects induced by current pharmacological approaches, it is necessary to create alternative therapies that have greater efficacy and bioavailability, and rarer side effects [12,13,14]. 

In this regard, natural products from plants or foods can represent a real source of new compounds with therapeutic value for neurodegenerative diseases. 

For many years, mushrooms have been used not only as food, but also in traditional medicine for their anticancer, antiviral, antibacterial, antioxidant and immunomodulatory effects [15,16,17]. Mushrooms have a diverse spectrum of bioactive substances that support their use in the preservation of human health or in the prevention of nervous disorders [18]. 

*Hericium erinaceus* is one of the most well-studied medicinal mushrooms, with an emphasis on the neurological system [19,20]. This mushroom’s anti-neuroinflammatory properties have been revealed in an increasing number of studies in recent years. 

A high-fat, high-sucrose diet improves spatial learning ability and decreases the synthesis of tumor necrosis factor-α (TNF-α) and interleukin-1 (IL-1) mRNA in the hippocampus, resulting in an anti-inflammatory effect in 15-month-old mice [21,22]. 

Another study discovered that administering *Hericium erinaceus* for 30 days reduced astrocyte and microglia activation in the cerebral cortex and hippocampus of AD APPswe/PS1dE9 transgenic mice. Furthermore, in an AD mice model, the anti-inflammatory action of *Hericium erinaceus* was found to diminish Aβ deposition, and enhance nerve growth factor (NGF) expression and hippocampal neurogenesis [23]. 

*Hericium erinaceus* has been shown to have antioxidant activity in addition to anti-inflammatory activity by working on the NF-E2-related factor 2 (Nrf2)/heme oxygenase 1 (HO-1) pathway [24,25]. As a result, *Hericium erinaceus* could be deemed an excellent neuroprotectant. 

Our latest research has demonstrated that by reducing oxidative stress and activating the NLRP3 inflammasome, this fungus can manage the typical abnormalities of AD, such as behavioral changes, phosphorylated Tau levels, aberrant overexpression of APP, β-amyloid formation and neuronal degeneration [26]. 

*Coriolus versicolor* is another fungus that has been intensively investigated in recent years and has been demonstrated to have a neuroprotective effect. According to recent research, this mushroom may have anticancer, anti-inflammatory, antioxidant, antibacterial and immunomodulatory effects [27,28,29,30]. 

*Coriolus versicolor*, like *Hericium erinaceus*, has been demonstrated to improve spatial memory in a mouse model of AD by enhancing antioxidant activity and inhibiting pro-inflammatory cytokines [28,31]. Furthermore, *Coriolus versicolor* treatment has been demonstrated to raise the levels of the proteins involved in the cellular stress response, such as HO-1, thioredoxin (Trx) and Nrf2 in the brain [32]. 

Another important aspect of *Coriolus versicolor* and *Hericium erinaceus* to consider is their ability to increase levels of the anti-inflammatory mediator lipoxin A4 (LXA4). According to our previous studies, this may be associated with the increased expression of the proteins involved in redox balancing [25,32]. 

Therefore, we hypothesize that the administration of *Coriolus versicolor* and *Hericium erinaceus* is able to prevent the neurodegenerative process in an animal model of PD induced by rotenone, a widely used fat-soluble insecticide. 

Rotenone can induce oxidative stress, neuroinflammation and dopaminergic cell death (DA) [33,34,35]. Furthermore, rotenone-induced neuropathological changes in the central nervous system are time-dependent and may also result in α-synuclein (α-syn) accumulation in the enteric nervous system and dorsal vagus motor nucleus, possibly through transneuronal and retrograde axonal transport. Because of rotenone’s wide neurotoxic effects, non-motor manifestations and signs such as gastrointestinal, olfactory, and cardiovascular problems, depression, sleep difficulties, cognitive loss and hyperalgesia might emerge before motor impairments [36].

Therefore, our idea is that the association between *Coriolus versicolor* and *Hericium erinaceus* activating the Nrf2/HO-1 redox system and, consequently, increasing the LXA4 levels is able to favor the resolution of oxidative stress and neuroinflammation and limit motor and non-motor symptoms characteristic of PD.

## 2. Materials and Methods

### 2.1. Animals

We utilized CD1 male mice weighing approximately 25–30 g (Envigo, Milan, Italy). They were first acclimated to their surroundings for a week before being placed in a precise environment (22 ± 1 °C with a 12 h dark, 12 h light cycle) with access to water and rodent food. Prior to the study’s execution, we obtained approval from the University of Messina’s Review Board for animal care. All animal tests were carried out in accordance with the latest Italian procedures (D.Lgs 2014/26), EU rules (EU Directive 2010/63) and the ARRIVE recommendations.

### 2.2. Hericium Erinaceus and Coriolus Versicolor Biomass Preparation

*Hericium erinaceus* (H) and *Coriolus versicolor* (C) biomasses, containing mycelium and primordia of the respective mushroom, were generously donated by Mycology Research Laboratories Ltd. (MRL, Luton, UK) as a commercially available product and were used for research. The preparation of the two mushrooms is described in the previous research of Trovato et al. [25,32]. The optimal dose (200 mg/kg) was chosen based on the dose used in human trials with cancer or HPV patients (3 g/day) [37], a regimen also verified by rat investigations. The characterization of the two fungi was performed via chromatography–Orbitrap–mass spectrometry (LC-Orbitrap-MS) and via gas chromatography–tandem mass spectrometry (GC-MS/MS) as earlier described, and the results of the characterization are visible in the work of D’Amico R. et al. [38]. 

### 2.3. Rotenone-Induced PD and Treatment

PD was induced by rotenone (Rot) (5 mg/kg in 4% carboxymethylcellulose (CMC); Sigma, USA) as previously described [39]. *Hericium erinaceus* (H) and *Coriolus versicolor* (C) were administrated orally at the dose of 200 mg/kg for 28 days and 1 h after Rot administration.

#### Experimental Group

Animals were casually distributed into the following groups (*n* = 15 mice for each group):

Group 1: Sham = vehicle solution (saline) was administrated quotidianly by oral gavage, as per Rot protocol.
Group 2: Sham+H = H (200 mg/kg) solution was administrated by oral gavage for 28 days (data not shown).
Group 3: Sham+C = C (200 mg/kg) solution was administrated by oral gavage for 28 days (data not shown).
Group 4: Sham+(H+C) = H+C (200 mg/kg) solution was administrated by oral gavage for 28 days (data not shown).
Group 5: Rot+vehicle = Rot solution was administrated quotidianly by oral gavage and vehicle solution (saline) was administrated orally for 28 days.
Group 6: Rot+H = Rot solution was given orally every day by gavage, whereas H solution was given orally for 28 days and 1 h after Rot administration.
Group 7: Rot+C = Rot solution was given orally every day by gavage, whereas C solution was given orally for 28 days and 1 h after Rot administration.
Group 8: Rot+(H+C) = Rot solution was administrated daily by oral gavage and H+C solution was administrated orally for 28 days and 1 h after Rot administration.

On the 28th day the mice were anesthetized, and the brains were taken for molecular and biochemical analyses. 

### 2.4. Behavioral Testing

Behavioral assessments were performed on animals (*n* = 5 for each group) 1 day before and 28 days after the Rot injection.

#### 2.4.1. Open Field (OF)

The OF test was used to measure spontaneous locomotor activity. The device was a 50 × 50 cm Plexiglas box with a floor divided into sixteen squares (four quadrangles were defined as the center and twelve quadrangles along the walls as the periphery). We executed the test as previously described [40]. Briefly, each mouse was positioned in the center of the box, and movement was filmed as a line crossing when the animal withdrew all four paws from one square to another. For 5 min, the number of crossings and time spent in the center were valued and recorded.

#### 2.4.2. Rotarod Test (RT)

The rotarod system was used to measure mice’s motor performance on a moving rod following the protocol described in our other studies [41]. In short, the mice were placed on a revolving rod (1 cm diameter for mice), and the rotation speed was gradually increased from 0 to 12,000× *g* in 60 s. The latency (the time a mouse spends on the rod) was measured. This was performed up to 5 times in one session (with a rest period that increased by 5 s with each fall).

#### 2.4.3. Catalepsy Test (CT)

Catalepsy was measured as previously described, with a diminished ability to initiate movement and a failure to modify posture [42]. Mice were placed with their hindquarters on the bench and their forelimbs on a horizontal bar 4 cm above the bench to study catalepsy. The mice’s time in this position was recorded using a timer set to 180 s. Cataleptic mice were those who remained in this position for 30 s or longer.

#### 2.4.4. Elevated Plus Maze (EPM) 

As previously explained, the EPM test was used to assess anxiety-like behavior [43]. The apparatus was made up of two open arms (30 × 5 × 0.25 cm) and two enclosed arms (30 × 5 × 15 cm) in black Plexiglas with a light gray floor that extended from a central platform (5 × 5 cm); the complete apparatus was elevated to a height of 60 cm above the floor level by a single central support. Concisely, the mouse was placed on the central platform and allowed to discover the maze for 5 min; the number of entries in each arm and the number of crossings were documented. Anxiety was indicated by a decrease in both the amount of time spent in the open arms and the proportion of admissions into the open arms.

#### 2.4.5. Tail Suspension Test (TST)

The TST was conducted to value depression-related behavior in mice. This behavioral test was carried out exactly as previously reported [44]. Briefly, the mice were suspended from their tails 50 cm above the surface using adhesive tape wrapped around the tail 2 cm from the tip. For 6 min, immobility (defined as the absence of any limb or body movements other than those caused by respiration or gravity) was measured.

#### 2.4.6. Sucrose Preference Test (SPT)

The SPT is used to measure anhedonia in mice. Two bottles were used for the test, one with water and one containing a 2% sucrose solution. Both bottles were weighed at the start of the test and were, subsequently, placed randomly. After 12 h, the position of the bottles was changed, and they were removed and reweighed after another 12 h (total test time of 24 h). To calculate the sucrose preference ratio, the following equation was performed:

Sucrose preference ratio (%) = [Sucrose intake (g)/(Sucrose intake (g) + Water intake (g))] × 100%

### 2.5. Histology

The brains of five mice in every group were fixed, sliced, and stained with hematoxylin and eosin (H&E, Bio-Optica, Milan, Italy), and the sections were evaluated using an optical microscope linked to an imaging system (Leica Microsystems SpA), as earlier described [34,45]. Histopathological valuation was carried out blindly using a semi-quantitative five point rating: 0 for a normal condition (no death neurons observed); 1 for an irrelevant condition (substantia nigra (SN) contained 1 to 5 death neurons); 2 for a modest condition (SN contained 5 to 10 death neurons); 3 for a severe condition (SN contained more than 10 death neurons); and 4 for a more severe condition (SN contained only death neurons) [46]. The scores from all slides were averaged to give a concluding score for each individual mouse. Images are demonstrative of all animals in every group.

### 2.6. Stereology 

Unbiased numbering of TH+ dopaminergic neurons inside the SN was carried out as previously described [47]. To summarize, mice were killed and their brains were extracted and post-fixed in 4% paraformaldehyde at 4 °C overnight. A vibratome was used to cut serial slides (40 μm thick) through the SN in the coronal plane from each block. Every fourth free-floating section was incubated overnight with anti-tyrosine hydroxylase (TH) (1:400, Millipore, Burlington, MA, USA) before being processed using the ABC technique (Vector Laboratories, Burlingame, CA, USA). Sections were cover-slipped and counterstained with cresyl violet, a Nissl stain. TH+ cells were TH immunoreactive, whereas TH- cells were not TH immunoreactive but were Nissl stained. The stereologist was completely unaware of the treatment received. Stereo Investigator software was used to evaluate five typical SNpc slices from each mouse brain (Microbrightfield, Williston, VT, USA).

### 2.7. Quantification of LXA4, IL-1β and TNF-α

The supernatant of the brain tissue homogenate was centrifuged and used [32,48,49]. LXA4, IL-1β and TNF-α were analyzed using an ELISA kit (Cloud-Clone Corp, Wuhan, China). The assay was performed following the instructions of the manufacturer and measured by a microplate reader at 450 nm.

### 2.8. Immunohistochemical Investigation for TH

The immunohistochemical techniques used have been previously described [50]. Sections of the midbrain were incubated overnight with the primary antibody: anti-TH (Millipore, 1:500 in PBS, *v*/*v*, AB152). The sections were then washed with PBS and treated with the secondary antibody the next day. A biotin-conjugated goat anti-rabbit IgG and an avidin–biotin–peroxidase complex were used to identify specific labeling (Vector). To test the antibodies’ specificity, brain slices from 5 mice in each group were treated with either a primary or secondary antibody. The photos were captured with a Leica DM6 camera (Leica Microsystems SpA, Milan, Italy) [51]. Densitometric analysis was performed using the ImageJ IHC profiler plug-in. When this option is chosen, it automatically creates a histogram profile of the analyzed DAB image and displays a score log [52]. The histogram profile matches to the computer program’s positive pixel intensity value [53]. Immunohistochemical analyses were carried out by experienced individuals who were unaware of the treatment.

### 2.9. Western Blot Analysis

Western blot analysis was carried out as previously described [54,55]. Primary antibodies used were: anti-dopamine transporter (DAT) (1:500; Santa Cruz Biotechnology, Dallas, TX, USA; (sc)), anti-α-syn (1:500; (sc)), anti-Nrf2 (1:500; (sc)), anti-HO-1 (1:500; (sc)), anti-Hsp-70 (1:500; (sc)), anti-IkB-α (1:500; (sc)), anti-nuclear factor-kB (NF-kB) p65 (1:1000; (sc)), anti-glial fibrillar acid protein (GFAP) (1:1000; (sc)), anti-ionized calcium-binding adaptor molecule 1 (Iba-1) (1:1000; (sc)), anti-Bax (1:500; (sc)), anti-Bcl-2 (1:1000; (sc)). The membranes were then incubated with IgG peroxidase-conjugated secondary antibody-conjugated bovine mouse or IgG peroxidase-conjugated goat anti-rabbit (1:2000, Jackson ImmunoResearch, West Grove, PA, USA). Probing the membranes with a β-actin or laminin antibody revealed the protein content. The manufacturer’s advanced chemiluminescence detection system reagent was used to detect the target antibody signals (SuperSignal West Pico Chemiluminescent Substrate, Pierce). The expression of the protein bands was determined by densitometry and adjusted to β-actin levels using BIO-RAD ChemiDocTM XRS Plus software. Blot signal images (8-bit resolution/600 dpi) were imported into the analysis application (ImageQuant TL, v2003) [56].

### 2.10. Statistical Analysis

The values in the figures and text are expressed as the average ± SD and are typical of at least three experiments performed at separate times. Unless otherwise stated, each experiment utilized 15 mice per group. A one-way analysis of variance was used to review the data, followed by a Bonferroni post hoc test for multiple comparisons. A *p*-value of less than 0.05 was considered statistically significant.

## 3. Results

### 3.1. Effects of H+C on Oxidative Stress

**Figure 1:**
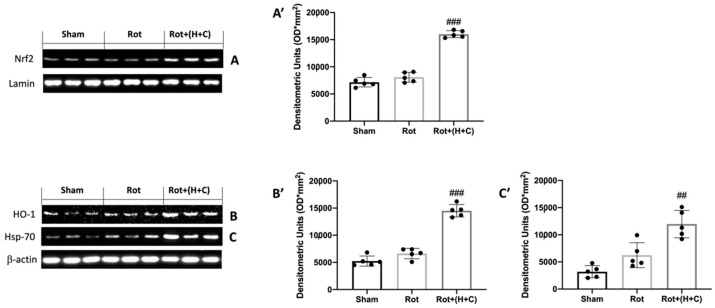
Effects of H+C on Nrf2, HO-1 and Hsp-70 after Rot intoxication. Western blot analysis revealed, compared with Sham group, a slight elevation of Nrf2 (**A**), HO-1 (**B**) and Hsp-70 (**C**) in Rot-treated mice. Animals subjected to treatment with H+C revealed a significant increase in these proteins. See the respective densitometric graphs (**A’**,**B’**,**C’**). Values are expressed as mean ± SD of three independent analyses on 5 animals/group. ## *p* < 0.01 compared to Rot; ### *p* < 0.001 compared to Rot.

Oxidative stress in the brain is associated with an increased expression of genes that contributes to free radical detoxification. In this regard, we wanted to evaluate, by way of Western blot analysis, the expression of proteins responsible for redox balance such as Nrf2 and HO-1, and, in addition, the expression of the chaperone Hsp-70, which ensures the stabilization of other protein molecules under various stresses. The results obtained showed a slight activation of the three proteins following the treatment with rotenone (Figure 1A,A’,B,B’,C,C’, respectively). The H+C treatment was able to further raise the levels of Nrf2 and HO-1 by regulating the redox imbalance, and also raise that of Hsp-70 (Figure 1A,A’,B,B’,C,C’, respectively).

### 3.2. Effects of H+C on LXA4 Levels and Glial Cell Activation

**Figure 2:**
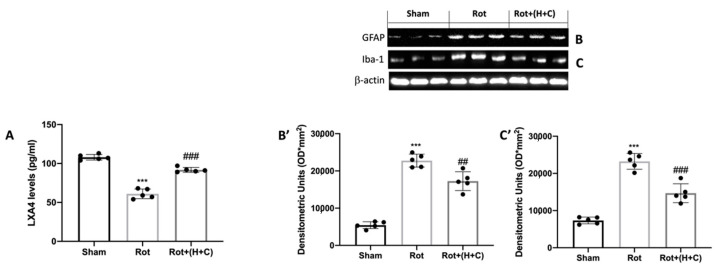
Effects of H+C on LXA4 levels and on astrocytes and microglia after Rot intoxication. LXA4 protein levels in midbrain were evaluated via ELISA kit in Sham, Rot and Rot+(H+C) groups (**A**). Western blot analysis revealed, compared with Sham group, an evident increase in GFAP (**B**) and Iba-1 (**C**) in Rot-treated mice. Animals subjected to treatment with H+C revealed a significant decrease in astrocytes (**B**) and microglia (**C**). See the respective densitometric graphs (**B’**,**C’**). Values are expressed as mean ± SD of three independent analyses on 5 animals/group. *** *p* < 0.001 compared to Sham; ## *p* < 0.01 compared to Rot; ### *p* < 0.001 compared to Rot.

LXA4 expression was studied in the brain taken from mice of the Sham, Rot and Rot+(H+C) groups. As shown in Figure 2A, the control animals showed baseline LXA4 levels, whereas a reduction was observed following rotenone intoxication. Administration of H+C for 28 days induced a significant increase in the LXA4 protein level (Figure 2A). Since LXA4 is important for the response to inflammatory processes, we wanted to evaluate the effect of H+C also on the activation of glial cells, such as astrocytes and microglia. The levels of GFAP and Iba-1 were very low in the Sham group, but significantly higher in the Rot-treated mice. The H+C treatment effectively reduced the elevated expression of GFAP and Iba-1 under these conditions (Figure 2B,B’,C,C’, respectively).

### 3.3. Effects of H+C on NF-kB Pathway and Pro-Inflammatory Cytokines

**Figure 3:**
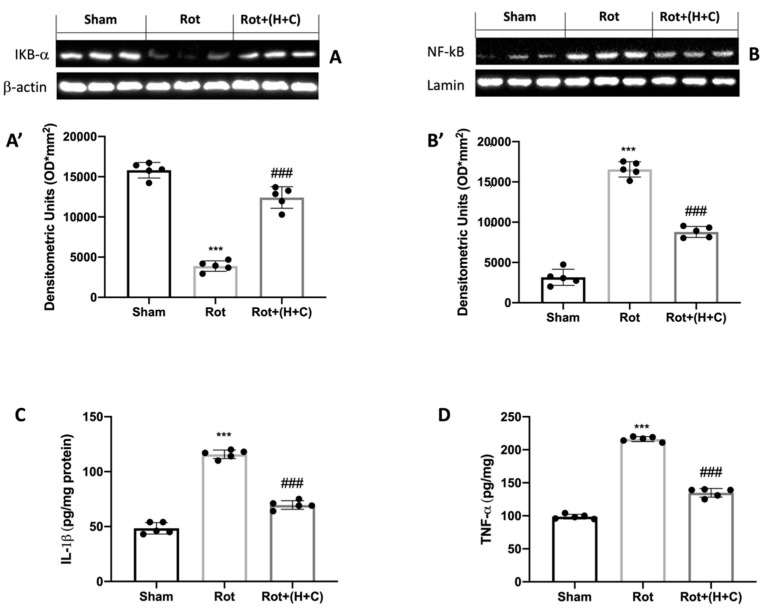
Effects of H+C on IkB-α, NF-kB, IL-1β and TNF-α after Rot intoxication. Western blot analysis revealed, compared with Sham group, an evident degradation of IkB-α (**A**) and nuclear translocation of NF-kB (**B**) in Rot-treated mice. Animals subjected to treatment with H+C revealed a significant restoration of these two proteins. See the respective densitometric graphs (**A’**,**B’**). IL-1β (**C**) and TNF-α (**D**) protein levels in midbrain were evaluated via ELISA kit in Sham, Rot and Rot+(H+C) groups. Values are expressed as mean ± SD of three independent analyses on 5 animals/group. *** *p* < 0.001 compared to Sham; ### *p* < 0.001 compared to Rot.

We used the Western blot analysis to look at the expression of NF-kB and IkB-α to see if H+C had an anti-inflammatory effect on the NF-kB pathway. Our findings showed increased IkB-α degradation in Rot-injured mice compared to Sham mice, whereas the treatment with H+C increased IkB-α cytosolic activity (Figure 3A,A’). As for NF-kB, an increase in nuclear translocation was observed in the rotenone group, but treatment with H+C attenuated the nuclear expression of this protein (Figure 3B,B’). Since cytokines are also downstream gene targets of NF-kB, we used ELISA kits to look at IL-1β and TNF-α levels (Figure 3C). When Rot-treated animals were compared to control mice, there was a significant increase in IL-1β levels, but H+C-treated mice had low levels of this cytokine. The cytokine TNF-α produced similar results (Figure 3D). 

### 3.4. Effect of H+C Treatment on Apoptosis 

**Figure 4:**
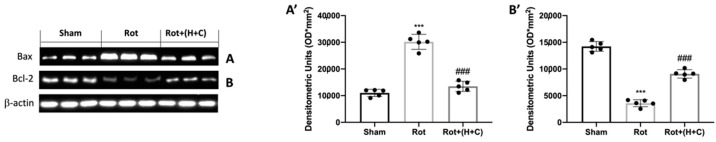
Effects of H+C on Bax and Bcl-2 expression after Rot intoxication. Western blot analysis demonstrated Bax expression to be significantly increased in the Rot group, whereas treatment with H+C significantly limited the rise in Bax expression (**A**). Bcl-2 expression was reduced after Rot intoxication; however, treatment with H+C restored the basal levels (**B**). See the respective densitometric graphs (**A’**,**B’**). Values are expressed as mean ± SD of three independent analyses on 5 animals/group. *** *p* < 0.001 compared to Sham; ### *p* < 0.001 compared to Rot.

To assess the effect of the H+C treatment on Rot-induced apoptosis, we used the Western blot technique to examine the expression of Bax and Bcl-2. Tissues from Rot-treated mice had higher levels of Bax expression than control mice. This expression was lowered by the H+C treatment (Figure 4A,A’). Samples removed from Sham mice exhibited baseline levels of Bcl-2, whereas the administration of Rot reduced this expression. The H+C treatment restored Bcl-2 expression to baseline levels (Figure 4B,B’).

### 3.5. Effects of H+C Treatment on Specific Markers of PD

**Figure 5:**
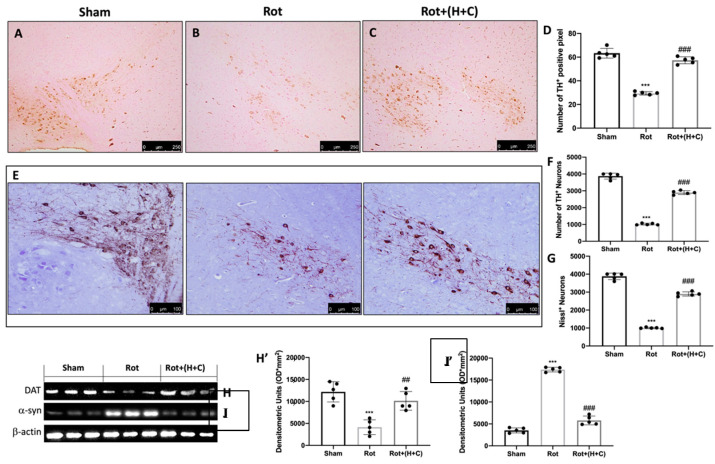
Effects of H+C on TH, DAT and α-syn expression in Rot-treated mice. The immunohistochemical analysis has shown, compared with the Sham mice (**A**), a noticeable loss of TH-positive cells in mice of the Rot group (**B**). Animals treated with H+C revealed an increase in TH expression (**C**). Data are expressed as % of TH-positive pixels and are the SD means of *n* = 5 mice/group (**D**). *** *p* < 0.001 compared to Sham; ### *p* < 0.001 compared to Rot. Stereological counting of TH+ and cresyl-violet-positive neurons in substantia nigra slices from one hemisphere (**F,G**). Images of TH immunohistochemistry in midbrain slices counterstained with cresyl violet (**E**). Each data point is expressed as the number of TH+ and Nissl+ neurons and is the mean SD from *n* = 5 mice per group. *** *p* < 0.001 when compared to Sham; ### *p* < 0.001 when compared to Rot. Western blot analysis revealed, compared with Sham group, an evident decrease in DAT in Rot-treated mice. Animals subjected to treatment with H+C revealed a significant rise in DAT (**H**). See densitometric graph (**H’**). In addition, this analysis revealed a significant increase in α-syn in the Rot group compared to Sham animals. The H+C treatment significantly reduced the increase in this protein (**I**). See densitometric graph (**I’**). The data are representative of at least three independent experiments and are expressed as the mean ± SD of *n* = 5 mice for each group. (**I**) *** *p* < 0.001 compared to Sham; ## *p* < 0.01 compared to Rot; ### *p* < 0.001 compared to Rot.

To demonstrate the effect of H+C treatment on the DA pathway, the expression of TH and DAT was measured. When compared to the control group (Figure 5A,D), the mice given only rotenone had a considerable reduction of TH-positive cells in the midbrain (Figure 5B,D). The H+C treatment, on the other hand, has been demonstrated to drastically inhibit the loss of TH-positive neurons in the midbrain (Figure 5C,D). Unbiased stereology of nigral TH+ neurons performed 28 days after Rot intoxication showed significant neuroprotection by the H+C treatment. When compared to the Sham group, the number of TH+ neurons in mice decreased considerably following Rot injection. Following the H+C treatment, this loss diminished (Figure 5E,F). Similarly, Rot lesioning drastically decreased Nissl-stained neurons, but not in mice treated with H+C. (Figure 5E,G). Furthermore, through Western blot analysis we found a significant decrease in DAT in Rot-injected animals compared to the Sham group, but the H+C treatment significantly restored DAT levels (Figure 5H,H’). α-syn is the most abundant component of Lewy bodies, which are intraneuronal protein aggregates. Because α-syn accumulation is a hallmark of PD, we wanted to assess its expression to establish H+C’s ability to prevent the neurodegenerative process. This protein was found to be much higher in Rot-injured animals than in the Sham group. Instead, after rotenone intoxication, the H+C treatment significantly reduced α-syn expression in the midbrain (Figure 5I,I’).

### 3.6. Effects of H or C or H+C on Histological and Behavioral Rot-Induced Alteration

**Figure 6:**
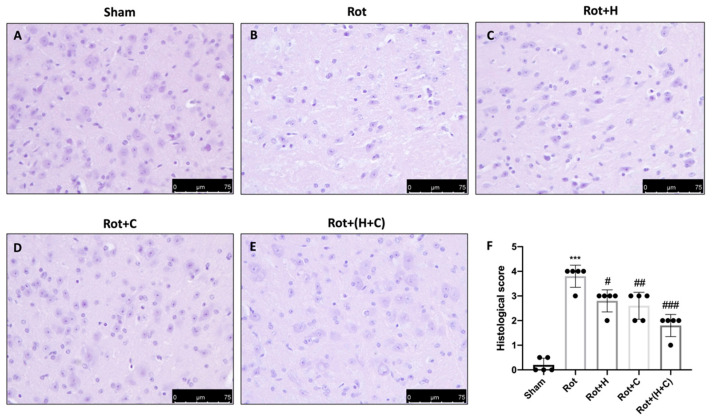
Effect of H or C or H+C on histological parameters in Rot-lesioned mice. Rot-injured mice had nigrostriatal dopaminergic degeneration, which translated into neuronal cell loss in the midbrain (**B**) in comparison to the normal neuronal cell structure seen in control mice (**A**); H (**C**), C (**D**) and, especially, H+C (**E**) treatment reduced alteration of the dopaminergic tract, mitigating neuronal cell loss. See histological score (**F**). The data are representative of at least three independent experiments and are expressed as the mean ± SD of *n* = 5 mice for each group. (**F**) *** *p* < 0.001 compared to Sham; # *p* < 0.05 compared to Rot; ## *p* < 0.01 compared to Rot; ### *p* < 0.001 compared to Rot.

H&E staining was conducted on brain sections to assess the histopathological changes caused by Rot treatment. Mice given saline for 28 days had a regular brain architecture and a usual number of neurons in the midbrain (Figure 6A,F). Instead, the Rot treatment caused cytoplasmic vacuolization and nigrostriatal neuronal cell death in mice. When Rot mice were compared to control mice, the brain architecture was altered (Figure 6B,F). In contrast, mice treated with H, C and, especially, H+C showed an evident reduction in cytoplasmic vacuolization and cell loss in the midbrain (Figure 6C–F). 

**Figure 7:**
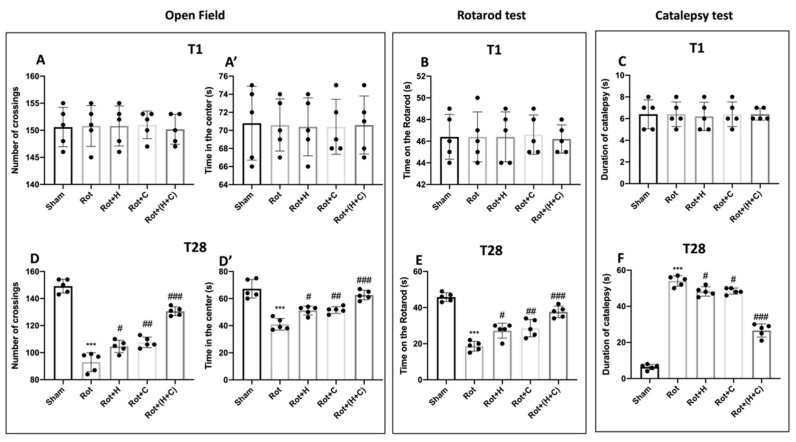
Effect of H or C or H+C on behavioral motor alterations in Rot-lesioned mice. Motor function was assessed using OF (**A**,**A’** for T1 and **D,D’** for T28), RT (**B** for T1 and **E** for T28) and CT (**C** for T1 and **F** for T28). The data are expressed as the mean ± SD of 5 mice for each group. *** *p* < 0.001 compared to Sham; # *p* < 0.05 compared to Rot; ## *p* < 0.01 compared to Rot; ### *p* < 0.001 compared to Rot.

**Figure 8:**
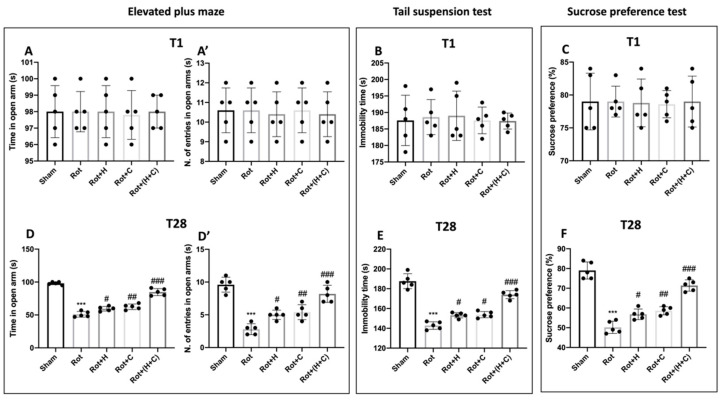
Effect of H or C or H+C on behavioral non-motor alterations in Rot-lesioned mice. The behavioral analysis of non-motor symptoms was assessed using EPM (**A**,**A’** for T1 and **D,D’** for T28), TST (**B** for T1 and **E** for T28) and SPT (**C** for T1 and **F** for T28). The data are expressed as the mean ± SD of 5 mice for each group. *** *p* < 0.001 compared to Sham; # *p* < 0.05 compared to Rot; ## *p* < 0.01 compared to Rot; ### *p* < 0.001 compared to Rot.

To evaluate the link between rotenone-induced dopaminergic neurodegeneration and recovery pathways, we examined motor action 1 day before and 28 days after rotenone induction. The data at time point 1 are not shown because there were no important differences between the groups (Figure 7 and Figure 8A,A’,B,C). The OFT was performed to evaluate locomotor activity. We found that the number of crosses was lower in Rot-treated mice than in control mice (Figure 7D). Similarly, Rot-treated mice showed a reduction in the time spent in the center compared to Sham mice (Figure 7D’). On the other hand, the mice treated with the single fungi and mostly those treated with the combination showed a restoration of motor activity with an increase in both the crossings and the time spent in the center of the square (Figure 7D,D’). The RT was used to test motor coordination. After 28 days of PD induction, Rot-treated mice showed severe motor incoordination, as evidenced by a decrease in time spent on the rotarod and an increase in the frequency of falls. Motor deficits in mice treated with H, C and, especially, H+C, on the other hand, were dramatically decreased (Figure 7E). Furthermore, Rot had a significant cataleptic effect in mice. In fact, the mice showed a considerable rise in cataleptic symptoms 28 days following Rot injection. Otherwise, daily H or C and, especially, H+C treatment considerably shortened the length of catalepsy caused by Rot (Figure 7F). After 28 days of Rot intoxication, the mice show not only motor alterations, but also signs of non-motor PD.

In the EPM test, mice were observed for anxious behavior. The behavioral test we conducted revealed a significant increase in time spent in open arms and the number of entries in open arms after treatment with H, C and, especially, H+C compared to the Rot group (Figure 8D,D’). The depression that often accompanies anxiety is another important non-motor feature of PD. The TST was used to measure depression-like behavior on day 28 after Rot intoxication. Compared to the Sham mice, these mice were much more immobile during the 6 min interval. Treatment with the single compounds and, even more, with the H+C association reduced the immobility time, leading to values comparable to the Sham group (Figure 8E). Furthermore, we assessed the sucrose preference, an essential test used to assess anhedonia, which is the primary symptom of depression. When compared to control mice, the Rot group consumed considerably less sugar. On the contrary, the sucrose consumption in mice treated with C, H and, especially, with H+C was significantly increased compared to the Rot group (Figure 8F). The histological and behavioral results were performed on all groups and the results obtained showed a greater effect of the association between H and C compared to the single compounds; for this reason, the other analyses report only the data relating to the association.

## 4. Discussion

Infections, toxins, metabolic stress, invasive injury and autoimmunity are all examples of stresses that can cause redox imbalances and neuroinflammation that promote the progression of PD [57,58]. Although the identification of mechanisms capable of resolving oxidative stress and the pro-inflammatory environment induced by PD pathology remains an active research topic, brain cells are known to have created response networks that detect and control different types of stress to adapt to environmental changes and survive different types of damage [59,60].

Consistent with this idea, there are integrated survival responses in the brain that are regulated by redox-dependent genes known as vitagens, which include sirtuins, thioredoxin, Nrf2 and HO-1. These proteins actively detect and control various types of stress and neuronal damage [61,62].

The vitagenic system is, therefore, emerging as a potential neurohormetic target for new cytoprotective interventions, such as for the prevention of neurodegenerative diseases [63]. In particular, emerging interest is focusing on the search for molecules capable of activating this system to minimize the devastating consequences associated with cell damage induced by free radicals, such as in neurodegeneration.

The antioxidant action of H. erinaceus and C. versicolor, two widely studied mushrooms, has been highlighted in numerous studies on pathologies affecting the nervous system. In particular, their beneficial effect was observed in AD [25,26,32].

Moreover, thanks to the action on antioxidant defense systems, such as Nrf2/HO-1, these two mushrooms seem to be able to act indirectly also on LXA4, a metabolic product of arachidonic acid, which is considered an endogenous “stop signal” for inflammation and which has been shown to have significant anti-inflammatory abilities in a variety of diseases such as nephritis, periodontitis, arthritis and inflammatory bowel disease [64]. Studies conducted in our laboratories have described, for the first time, the distribution of the LXA4 protein in the brain. In particular, it has been shown that regions of the brain such as the substantia nigra and the septum are those with the lowest content of this neuroprotective agent. This finding is relevant for the pathogenesis of AD and PD. An even more remarkable finding is the ability of both C. versicolor and H. erinaceus to increase LXA4 levels in most regions examined [25,32].

Therefore, our study aimed to demonstrate that the association between C. versicolor and H. erinaceus acting on the Nrf2/HO-1 pathway is able to prevent oxidative stress induced by rotenone and, in this way, is able to resolve also the neuroinflammatory processes, probably through indirect action on LXA4 levels. According to our predictions, this action by the H+C compound will limit the accumulation of α-syn, the death of dopaminergic neurons and the behavioral disturbances associated with PD.

Nrf2, a transcription factor that activates many genes with cytoprotective functions, is involved in antioxidant and anti-inflammatory reactions [14,65,66,67]. These cytoprotective genes encode a wide variety of phase II detoxification enzymes, such as HO-1, glutathione S-transferase (GST), glutamate cysteine ligase, thioredoxin, thioredoxin reductase and many more [68,69,70]. Our results showed an increase, albeit insignificant compared to the controls, in the expression of important vitagens such as Nrf2 and HO-1 in rotenone-treated mice. The same trend was observed for Hsp-70, a protein important for the stabilization of other protein molecules in response to various stressors. Oral treatment with H+C has, instead, further increased the levels of these proteins, allowing for a more effective response to oxidative stress induced by rotenone.

Furthermore, in line with the results obtained previously, we showed that the control animals had basal levels of LXA4, whereas the treatment with the combination of the two mushrooms led to an increase in these levels, which were significantly decreased in the animals treated with rotenone, probably due to neuronal degeneration induced by oxidative stress.

At this point, knowing that the increase in LXA4 is important for the resolution of neuroinflammation, we evaluated the effect of H+C on the activation of glial cells such as astrocytes and microglia, which play important homeostatic roles in the central nervous system (CNS) by maintaining balancing neurotransmitters and extracellular ions and by providing nourishment [71,72,73]. However, the chronic malfunction or activation of these important cells can have negative consequences, culminating in neuroinflammatory cascades that initiate or worsen the pathogenic pathways that lead to neurodegeneration [74,75,76,77]. Our results demonstrated a significant increase in GFAP and Iba-1 in rotenone-treated mice, whereas those treated with the combination showed significantly less activation of both cell types.

The activation of astrocytes and microglia is linked to the release of a series of proinflammatory and neurotoxic proteins such as TNF-α and IL-1β, and with the activation of the NF-kB pathway [78,79]. Here, we have shown that the H+C association is able to prevent the nuclear translocation of NF-kB, a transcription factor responsible for the inflammatory response, and the degradation of IkB-α, thus limiting the release of pro-inflammatory cytokines, mediators and immunoregulators such as IL-1α and TNF-α.

Once it was demonstrated that the H+C association acted on oxidative stress and on the neuroinflammatory processes induced by rotenone, we wanted to evaluate the effect of H+C on apoptosis. In this regard, our results suggest that H+C protects neuronal cells from apoptosis as it drastically reduces the levels of the pro-apoptotic marker Bax by increasing the levels of the anti-apoptotic protein Bcl-2.

Furthermore, by evaluating two specific markers for dopaminergic neurons such as TH and DAT, we demonstrated how the administration of H+C during the entire duration of the experiment limited the death of these neurons, which is typical of PD.

Another important result that we have found following the administration of H+C is the reduction of α-syn, which generally aggregates and accumulates in dopaminergic neurons in subjects with PD. The combination of all these beneficial actions of H+C on oxidative stress, neuroinflammation and apoptosis of dopaminergic neurons has managed to reduce the motor and non-motor disorders induced by rotenone.

## 5. Conclusions

In conclusion, our results have shown that the H+C association is a very effective antioxidant and a powerful anti-inflammatory agent. Consequently, H+C is a beneficial nutritional supplement that could be employed as a preventive agent in the neurodegenerative process associated with PD. However, further studies are needed to clarify how Nrf2 is activated and how this pathway relates to LXA4.

## Data Availability

Based upon the rules of our laboratory, the datasets used in the current study are available from the corresponding author (dimpellizzeri@unime.it) on reasonable request.

## Abbreviations

NF-E2-related factor 2 (Nrf2); heme oxygenase 1 (HO-1); Parkinson’s disease (PD); Alzheimer’s disease (AD); tumor necrosis factor-α (TNF-α); interleukin-1 (IL-1); nerve growth factor (NGF); thioredoxin (Trx); lipoxin A4 (LXA4); α-synuclein (α-syn); Hericium erinaceus (H); Coriolus versicolor (C); chromatography–Orbitrap–mass spectrometry (LC-Orbitrap-MS); gas chromatography–tandem mass spectrometry (GC-MS/MS); rotenone (Rot); carboxymethylcellulose (CMC); Open Field (OF); Rotarod Test (RT); Catalepsy Test (CT); Elevated Plus Maze (EPM); Tail Suspension Test (TST); Sucrose Preference Test (SPT); substantia nigra (SN); tyrosine hydroxylase (TH); dopamine transporter (DAT); nuclear factor-kB (NF-kB); glial fibrillar acid protein (GFAP); ionized calcium-binding adaptor molecule 1 (Iba-1); glutathione S-transferase (GST).
